# Edge-level multi-constraint graph pattern matching with lung cancer knowledge graph

**DOI:** 10.3389/fdata.2025.1546850

**Published:** 2025-02-10

**Authors:** Houdie Tu, Lei Li, Zhenchao Tao, Zan Zhang

**Affiliations:** ^1^School of Artificial Intelligence, Hefei University of Technology, Hefei, China; ^2^Key Laboratory of Knowledge Engineering with Big Data (the Ministry of Education of China), Hefei University of Technology, Hefei, China; ^3^School of Computer Science and Information Engineering, Hefei University of Technology, Hefei, China; ^4^Department of Radiation Oncology, The First Affiliated Hospital of USTC, Division of Life Sciences and Medicine, University of Science and Technology of China, Hefei, China; ^5^Department of Radiation Oncology, Anhui Provincial Cancer Hospital, Hefei, China

**Keywords:** graph pattern matching, probability graph, lung cancer knowledge graph, Monte Carlo method, multi-constranint

## Abstract

**Introduction:**

Traditional Graph Pattern Matching (GPM) research mainly focuses on improving the accuracy and efficiency of complex network analysis and fast subgraph retrieval. Despite their ability to return subgraphs quickly and accurately, these methods are limited to their applications without medical data research.

**Methods:**

In order to overcome this limitation, based on the existing research on GPM with the lung cancer knowledge graph, this paper introduces the Monte Carlo method and proposes an edge-level multi-constraint graph pattern matching algorithm TEM with lung cancer knowledge graph. Furthermore, we apply Monte Carlo method to both nodes and edges, and propose a multi-constraint hologram pattern matching algorithm THM with lung cancer knowledge graph.

**Results:**

The experiments have verified the effectiveness and efficiency of TEM algorithm.

**Discussion:**

This method effectively addresses the complexity of uncertainty in lung cancer knowledge graph, and is significantly better than the existing algorithms on efficiency.

## 1 Introduction

Graph pattern matching (GPM) has always been crucial in graph computing, evolving to meet the requirements of emerging applications. The field of graph pattern matching was originally rooted in protein isomorphism research (Hu and Ferguson, 2016; Tian and Patel, 2008) and later expanded to cover community discovery (Liu et al., 2021; Su et al., 2022), expert identification (Li et al., 2016; Wei et al., 2014), development of recommender systems (Fan et al., 2013), social group discovery (Khan et al., 2020; Sato et al., 2016; Chikhaoui et al., 2020), and range group identification (Fan et al., 2010). In 2024, Li et al. Li et al. (2024) introduced the concept of probability graph pattern matching for lung cancer knowledge graph, and proposed a multi-constraint graph pattern matching algorithm TKG-McGPM, which combined with Monte Carlo method and candidate node screening to improve the diversity and validity of matching results. However, we believe there are some disadvantages that: (1) Different parameter characteristics: The node trust value *T*_*DT*_ is based on explicit relationships or attributes, and it is not suitable for the Monte Carlo method, while the marginal parameter diagnosis and treatment cycle value *T*_*DC*_ and the cost-benefit analysis value *T*_*CV*_ involve uncertainty factors, and they are more suitable for the Monte Carlo method. (2) Computational complexity and resource consumption: Monte Carlo methods require a lot of computational resources and are suitable for edge matching rather than node matching, as the latter tends to be more efficient and of lower complexity.

Therefore, it is more reasonable and efficient to apply the Monte Carlo method to edge matching. Hence, this paper proposes an edge-level multi-constraint graph pattern matching algorithm (TEM) based on lung cancer knowledge graph, which ensures the correctness of the matching results and increases the diversity by using the random method to obtain the values of the two parameters *T*_*DC*_ and *T*_*CV*_ on the edge. In order to further discuss the effectiveness and efficiency of using the Monte Carlo method on both nodes and edges, a hologram multi-constraint pattern matching algorithm (THM) has been proposed. Experimental results show that the TEM algorithm is superior to the existing algorithms and the THM algorithm. All in all, contributions of this paper include:

- The necessity of employing Monte Carlo methods for edge matching to achieve superior subgraph matches is proposed;
- To ensure optimal pattern graph alignment, the conventional graph pattern matching model is refined, introducing an edge-level approach tailored specifically to a lung cancer knowledge graph;
- Addressing prevailing challenges, we propose TEM, an edge-level graph pattern matching algorithm grounded in the context of a lung cancer knowledge graph;
- To rigorously assess the efficacy and efficiency of the TEM algorithm, we further introduce THM, a hologram pattern matching algorithm also rooted in the lung cancer knowledge graph framework.

## 2 Preliminary

### 2.1 Lung cancer knowledge graph

**Figure 1:**
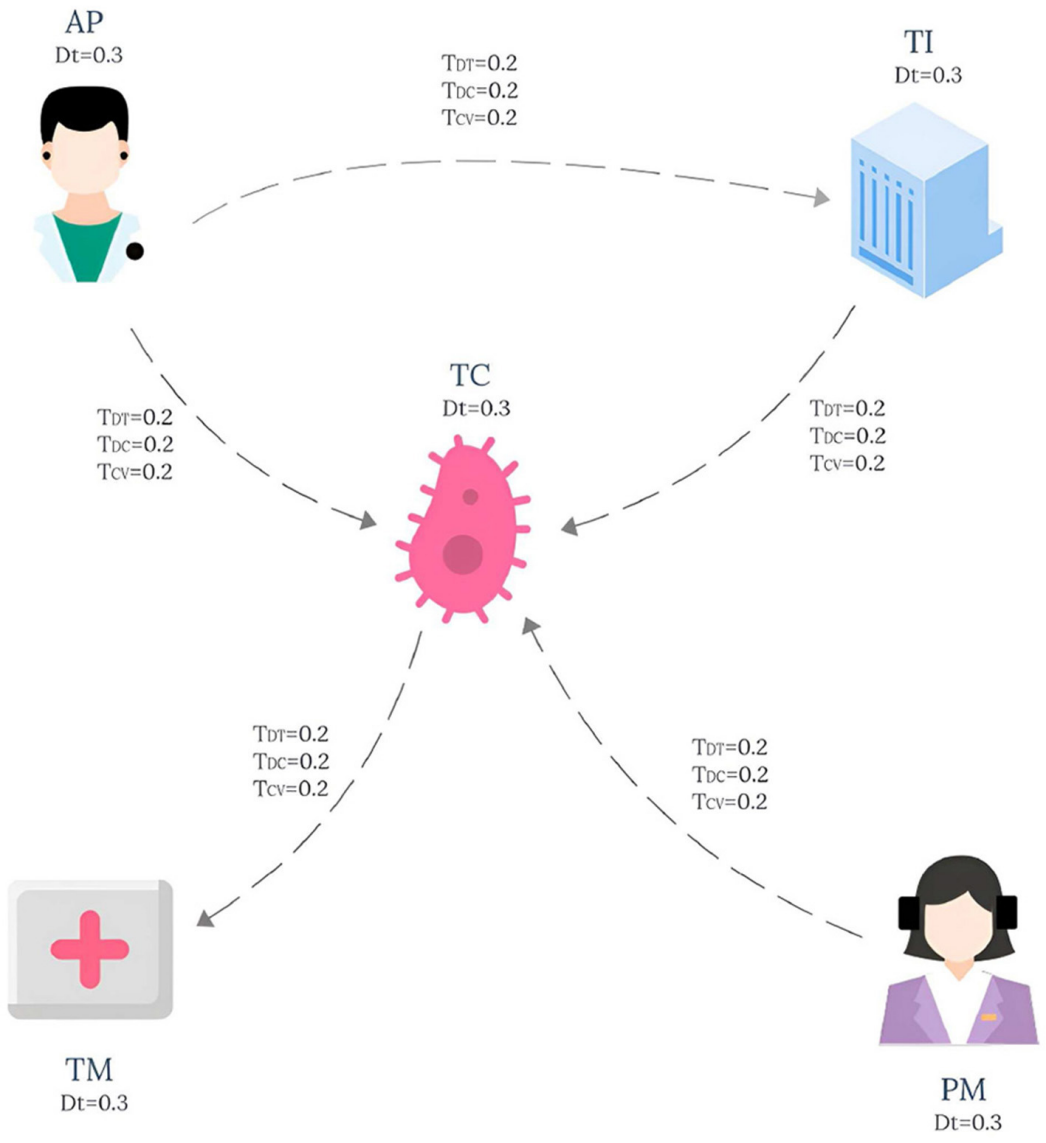
Pattern graph.

**Figure 2:**
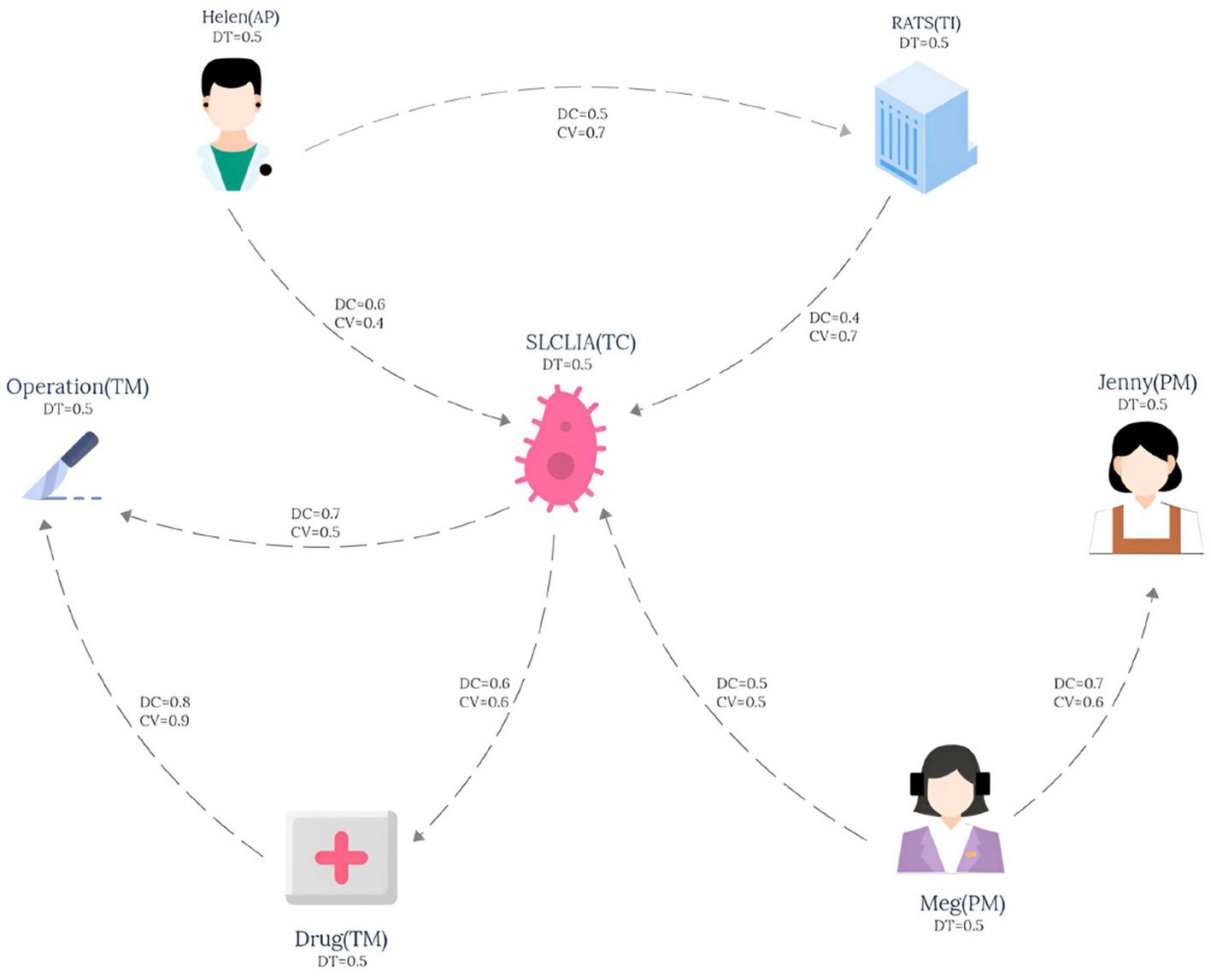
Data graph.

The knowledge graph used in this paper is derived from the tumor knowledge graph designed by Li et al. (2024) which consists of five participating nodes: attending physician (AP), testing instrument (TI), tumor type (TC), nursing staff (PM) and treatment method (TM). The pattern graph is shown in Figure 1 and the data graph is shown in Figure 2, where each node has an associated trust value reflecting its trustworthiness in the eyes of others.

The relevant concepts are defined as follows:

*Definition 2-1*: *Diagnostic and therapeutic trust value* *T*_*DT*_ represents the degree of trust between participating nodes in the tumor knowledge graph, ranging from 0 to 1.

*Definition 2-2*: *Diagnosis and treatment cycle value* *T*_*DC*_ represents the efficiency of resource allocation among these nodes, also in the range of 0 to 1.

*Definitions 2-3*: *Diagnostic and therapeutic cost-benefit analysis value* *T*_*CV*_ compares the cost of a medical intervention with its outcomes (e.g., survival and quality of life) to determine which intervention provides the highest cost-benefit ratio. There are two ways to calculate it: (1) The first approach (C/E) compares the effect under fixed costs and focuses on cost savings. It calculates the cost per unit of effect, such as the amount spent each year to extend life. This approach emphasizes cost savings. (2) The second approach (E/C) compares the effect of fixed costs and emphasizes the improvement in survival. It determines the effect generated per unit cost, considering the case of how a particular cost prevents multiple infection-related complications. This approach emphasizes improving survival. In this study, we assume that patients prioritize improved survival. Therefore, the patient chooses the second calculation method *T*_*EC*_. We compute *T*_*DC*_ and *T*_*CV*_ along the path by multiplication and *T*_*DT*_ by averaging.

### 2.2 Pattern graph matching

According to the correspondence between the data graph and the pattern graph, the graph pattern matching study can be divided into two categories: isomorphic GPM and simulated GPM. Isomorphic pattern matching requires a double-shot function to ensure that the topology of the matching subgraph perfectly reflects the pattern graph. Typical algorithms include VF2 (Foggia et al., 2001), VF3 (Carletti et al., 2018), R-join (Cheng et al., 2008), and G-Ray (Tong et al., 2007). This type of matching is key in 3D object matching and protein structure matching, and indexing, parallelization, and distribution methods are often used to improve efficiency. Due to its NP integrity, the computationally high cost makes the strict matching standard unsuitable for applications where accuracy is not a primary consideration. Based on this, scholars turned to simulation-based graph pattern matching. The concept of graph simulation was first proposed by Henzinger et al. (1995), and it requires that the nodes in the matching subgraph maintain the same successor relationship as their corresponding nodes in the pattern graph. Fan et al. (2010) converted exact one-to-one matching into binary relational search by bounded length, and Liu et al. (2015) extended bounded simulation to accommodate multi-constraint graph pattern matching, combining node and edge attribute information. Liu et al. (2020) proposed the multi-fuzzy constraint graph pattern matching to solve the limitation of ignoring the precursor adjacency relationship of the existing model, and introduced the strong simulation matching model of multi-fuzzy constraints, which matched the precursor relationship and the successor relationship of the candidate nodes at the same time, and effectively eliminated the nodes that did not meet the adjacency relationship. In addition, Liu et al. (2022) proposed a graph pattern matching model, which considers the number of nodes matched by each node in a fixed pattern graph, which is especially important when the matching subgraph contains too many matching nodes. For semi-supervised graph pattern matching with multiple constraints, Yan (2023) proposed a semi-supervised graph pattern matching algorithm based on bisimulation edge sequence guidance named DS-ES-SS GPM, which added the preference of decision makers to the matching process. Jin et al. (2023) proposes a strong simulation matching algorithm TPC-GPSSM based on timing priority constraint. The algorithm adds time order constraint in the matching process of the graph topology structure of the pattern graph to achieve the purpose of pruning in advance and reducing the computational complexity. Guo (2024) used graph pattern matching technology to predict the quality problems of the slab caused by the fluctuation of the characteristic mode of working conditions in the continuous casting process in real time, so as to provide guidance for the quality improvement of the slab.

**Figure 3:**
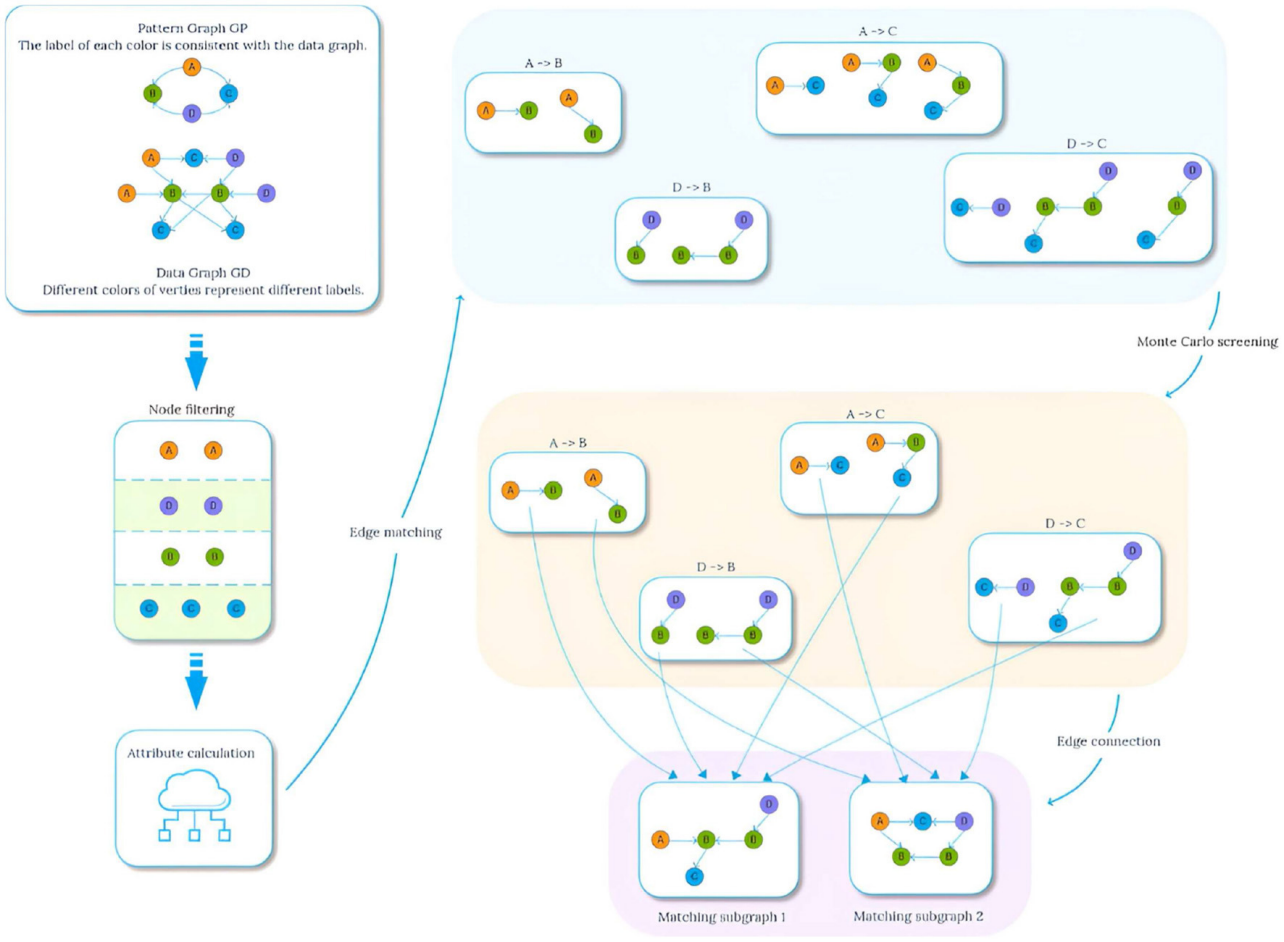
Matching process.

In order to study the application effect of graph pattern matching in medical field, Li et al. (2024) introduced the concept of probability graph pattern matching specially applicable to lung cancer knowledge graph, and proposed a multi-constrained graph pattern matching algorithm TKG-McGPM that combines Monte Carlo method and candidate node screening, aiming to enhance the diversity and effectiveness of matching results and assist patients in selecting the best tumor treatment plan. However, although the TKG-McGPM algorithm improves the matching performance, it still faces the challenge of balancing computational efficiency and accuracy when dealing with complex graph structures. Especially in the parameter processing of edges, how to effectively use the Monte Carlo method for optimization has become a key problem. Based on this, we propose the Edge Standardized Monte Carlo Matching Method (EdgeNormMC) to discuss the effect of applying the Monte Carlo method to the two parameters of the edge. In this method, the values of the two parameters *T*_*DC*_ and *T*_*CV*_ on the edges are scaled to the interval [0,1], and then the Monte Carlo method is used to perform multiple random sampling in the specified interval to select the optimal candidate edges from the possible matching set. The whole matching process is shown in Figure 3.

*Definition 2-4:* A data graph $G_{D}=\left(V,E,f_{V}^{D},f_{E}^{D}\right)$ is a directed graph with node and edge attributes, where:

- *V* is the set of nodes of the data graph;
- *E* is the set of edges of the data graph, and (*v*_*i*_, *v*_*j*_) ∈ *E* represents the directed edge from node *v*_*i*_ ∈ *V* to node *v*_*j*_ ∈ *V*;
- $f_{V}^{D}$ is a function defined on a set of nodes, and in a Medical Knowledge Graph (TKG), each node has an attribute constraint value *T*_*DT*_ and a label ρ, where ρ represents the type of node, and the value of ρ can be *AP*, *TI*, *TC*, *PM* and *TM*;
- $f_{E}^{D}$ is a function defined on a set of edges, and ∀*e* ∈ *E*, $f_{E}^{D}\left(v_{i},v_{j}\right)$ is the attribute set of *e*. In a TKG, for the directed edge (*v*_*i*_, *v*_*j*_), $f_{E}^{D}\left(v_{i},v_{j}\right)$ contains *T*_*DT*_, *T*_*DC*_ and *T*_*CV*_.

*Definition 2-5:* A pattern graph $G_{P}=\left(V_{P},E_{P},f_{V}^{P},f_{E}^{P},f_{l}^{P},f_{m}^{P}\right)$ is a directed graph with node and edge attributes, where:

- *V*_*P*_ is the set of nodes of the pattern graph;
- *E*_*P*_ is the set of edges of the pattern graph, and (*u*_*i*_, *u*_*j*_) ∈ *E*_*P*_ represents the directed edge from node *u*_*i*_ ∈ *V*_*P*_ to node *u*_*j*_ ∈ *V*_*P*_;
- $f_{V}^{P}$ is a function defined on *V*_*P*_, and ∀*u* ∈ *V*_*P*_, $f_{V}^{P}\left(u\right)$ is the attribute set of *u*. In TKG, the function $f_{V}^{P}\left(u\right)$ corresponding to node *u* has the same meaning as the attribute of the node set in the data graph above;
- $f_{E}^{P}$ is a function defined on *E*_*P*_, and ∀*e* ∈ *E*_*P*_, $f_{E}^{P}\left(e\right)$ is the attribute set of *e* such that for each edge in *E*_*P*_, and $f_{E}^{P}\left(u_{i},u_{j}\right)$ is the set of properties associated with (*u*_*i*_, *u*_*j*_).
- $f_{l}^{P}$ is a function defined on *E*_*P*_, and ∀(*u*_*i*_, *u*_*j*_) ∈ *E*_*P*_, $f_{l}^{P}\left(u_{i},u_{j}\right)$ is the length constraint of the edge (*u*_*i*_,*u*_*j*_), whose values are positive integers *k* or symbols *, respectively, indicating that the interval of length *u*_*i*_ to *u*_*j*_ of the edge does not exceed *k* or there is no length limit. In TKG, without loss of generality, $f_{l}^{P}\left(u_{i},u_{j}\right)=2$.
- $f_{m}^{P}$ is a set of membership constraint functions defined on node properties or edge properties.

*Definition 2-6 (Edge normalized Monte Carlo matching method, EdgeNormMC):* For a pattern graph $G_{P}=\left(V_{P},E_{P},f_{V}^{P},f_{E}^{P},f_{l}^{P},f_{m}^{P}\right)$ and a data graph $G_{D}=\left(V,E,f_{V}^{D},f_{E}^{D}\right)$, *G*_*D*_ matches *G*_*P*_, denoted as *G*_*P*_⊴*G*_*D*_, if there is a binary relationship S ⊆*V*_*P*_×*V*:

- For all *u* ∈ *V*_*P*_, there is *v* ∈ *V* such that (*u, v*) ∈ *S*;
- For each pair (*u, v*) ∈ *S*,
  - If there is a membership calculation function for node attribute in $f_{m}^{P}$, then the corresponding attribute in $f_{V}^{D}\left(v\right)$ only needs to satisfy the corresponding membership constraint; otherwise, $f_{V}^{D}\left(v\right)$ needs to satisfy the constraint $f_{V}^{P}\left(u\right)$ defined on node u;
  - *u*~*v*, $f_{V}^{P}\left(u\right)=f_{V}^{D}\left(v\right)$, and
  - For each edge (*u, u*′), from the data graph *G*_*D*_, there is a path *p* from *v* to *v*′, so that (*u*′, *v*′) ∈ *S*, $f_{V}^{P}\left(u^{\prime }\right)=f_{V}^{D}\left(v^{\prime }\right)$ and if $f_{l}^{p}\left(u,u^{\prime }\right)=k$, then the length of the interval from node *v* to node *v*′ in the path *plen*(*p*) ≤ *k*;
  - If there is a membership calculation function for the aggregated attributes on the matching path in $f_{m}^{P}$, the corresponding aggregated attributes in the edge only need to satisfy the corresponding membership constraints; otherwise, the aggregated attributes on the matching path need to satisfy the corresponding attribute constraints in $f_{E}^{P}$.

Example 2.1. Consider a lung cancer diagnosis and treatment plan that needs to be consulted, and the plan needs to be organized by five nodes: Attending physician (AP), Testing instruments (TI), Types of lung cancer (TC), paramedic (PM), and Treatment (TM), and the interaction between them is shown in **Figure 1**. The data graph can be expressed as $G_{D}=\left(V,E,f_{V}^{D},f_{E}^{D}\right)$, where represents the role type, role name and trust impact factor *T*_*DT*_, and $f_{E}^{D}$ represents the diagnosis and treatment cycle value *T*_*DC*_ and the diagnosis and treatment cost-benefit analysis value *T*_*CV*_ between participants. The pattern graph can be expressed as $G_{P}=\left(V_{P},E_{P},f_{V}^{P},f_{E}^{P},f_{l}^{P},f_{m}^{P}\right)$, where $f_{V}^{P}$ represents the role constraint and trust impact factor constraint *T*_*Dt*_ for pattern nodes, $f_{E}^{P}$ represents the diagnosis and treatment trust constraint *T*_*DT*_, diagnosis and treatment cycle value constraint *T*_*DC*_, diagnosis and treatment cost benefit analysis value constraint *T*_*CV*_ for matching path of pattern edges, and $f_{l}^{P}$ represents the matching path length constraint for pattern edges. $f_{m}^{P}=(f_{Dt}^{m},f_{DT}^{m},f_{DC}^{m},f_{CV}^{m},T_{Dtm},T_{DTm},T_{DCm},T_{CVm})$ *T*_*DTm*_, *T*_*DCm*_, *T*_*CVm*_), where $f_{DT}^{m}$ represents the membership calculation function defined on the node trust impact factor constraint *T*_*Dt*_, and *T*_*Dtm*_ represents the corresponding membership constraint value. $f_{DT}^{m}$,$f_{DC}^{m}$ and $f_{CV}^{m}$ respectively represent the membership functions defined on the pattern edge attribute constraints *T*_*DT*_, *T*_*DC*_ and *T*_*CV*_, while *T*_*DTm*_, *T*_*DCm*_ and *T*_*CVm*_ respectively represent the membership constraint values of the corresponding attribute constraints.

For the convenience of calculation, The membership constraint values *T*_*DT*_, *T*_*DC*_ and *T*_*CV*_ of each attribute are set to 0.2, and *T*_*Dtm*_ is set to 0.3. In this study, the main purpose of specifying the uniform value of the constraint value is to simplify the calculation process, so that readers can understand the matching calculation process more clearly and smoothly. It should be pointed out that in the actual correlogram, the values corresponding to each edge are not uniform, but show different situations, and these values are randomly generated. According to the EdgeNormMC definition, we can get the matching node Helen of AP in the pattern graph, because the outgoing edge (AP,TC) of AP matches the path(Helen,SLCLIA) in the data graph, and the outgoing edge (AP,TI) matches the path(Helen,RATS) in the data graph. The matching node SLCLIA of TC is determined because we can get that the incoming edge (AP,TC) of TC matches the path(Helen,SLCLIA) in the data graph, the incoming edge (TI,TC) matches the path(RATS,SLCLIA) in the data graph, and the incoming edge (AP,TC) matches the path(Meg,SLCLIA) in the data graph. We can get results similar to those of other matching nodes in the model, the final matched subgraph *G*_*sub*_ = (*V*_*sub*_, *E*_*sub*_), including *V*_*sub*_ = (Helen, RATS, SLCLIA, Operation, Drug, Meg). *E*_*sub*_=(Helen,RATS), (Helen,SLCLIA), (RATS,SLCLIA), (SLCLIA,Operation), (SLCLIA,Drug), (Drug,Operation), (Meg,SLCLIA).

## 3 GPM with lung cancer knowledge graph

At present, there are two main types of multi-constraint graph pattern matching algorithms. One is composed of two core modules, that is, the matching of pattern edges and the connection of matching paths based on the topology of the pattern graph. The other is based on sequential exploration of the topology of pattern nodes. The existing graph pattern matching algorithm in lung cancer domain, TKG-McGPM, adopts the matching algorithm NTSS based on topological ordered exploration of pattern nodes, and introduces Monte Carlo method in the node matching process, which has achieved certain results. However, considering that node matching focuses more on certainty and accuracy, while edge matching is more suitable for dealing with uncertainty and probabilistic simulation optimization, we believe that Monte Carlo method applied to edge matching may be more reasonable and efficient.

To solve the above problems, we first propose an edge-level graph pattern matching algorithm TEM based on lung cancer knowledge graph by using Monte Carlo method on edges. Experimental results show that the proposed algorithm is significantly better than the existing TKG-McGPM algorithm in performance. In order to further explore the application potential of Monte Carlo method in lung cancer graph pattern matching, we also propose a hologram pattern matching algorithm THM based on lung cancer knowledge graph. In this section, the main algorithm flow of the TEM algorithm will be detailed, and the THM algorithm will be introduced in the next section.

### 3.1 Description of the TEM algorithm

The matching process of pattern nodes is divided into two key stages. Firstly, according to the constraints on the pattern nodes in the pattern graph, the candidate nodes that meet the conditions are selected. Then, according to the topological structure characteristics of the pattern nodes in the pattern graph, the selected candidate nodes are further filtered.

In the lung cancer knowledge graph matching described in this paper, the constraints on the nodes include the constraint *label*_*v*_ on the node label and the constraint *T*_*Dt*_ on the node trust impact factor. In addition, $f_{m}^{P}$ also contains a membership calculation function $f_{Dt}^{m}$ for node trust impact factor constraint *T*_*Dt*_ and the corresponding membership constraint *T*_*Dtm*_. For a pattern node *u*, we have *label*_*v*_(u) ⊂ *label*_*v*_(*v*) and $f_{Dt}^{m}\left(Dt\right)$ ≥ *T*_*Dtm*_ if there exists a candidate node *v* ∈ *V*, and the set *Cand*_*u*_ of candidate nodes of *u* can be obtained by the GetNodeCandidate method as shown in Algorithm 1.

**Algorithm 1:** GetNodeCandidate Algorithm for pseudo code

Require: Node whose mode is to be matched u ∈ *V*_*P*_, Node set V of the data graph
Ensure: The set of candidate nodes *Cand*_*u*_ of *u*
while For each node V in v, if V.visited =false do
if *label*_*v*_(u) ∈ *label*_*v*_(v) and $f_{D}^{m}t\left(Dt\right)$ ≥ Dtm then
Add *v* to *u*'s set of candidate nodes set *Cand*_*u*_
end if
end while
return *Cand*_*u*_

The TEM algorithm proposed in this paper still uses the node topology order to match pattern nodes, and this method can prune invalid matches by judging whether the candidate nodes meet the previous topology structure.

The input of the TEM algorithm is data graph *G*_*D*_ and pattern graph *G*_*P*_, and the output is the set $G_{sub}^{All}$ of matching subgraphs. Firstly, a topologically ordered sequence *V*_*T*_ of pattern nodes and a node *V*_*E*_ with indegree 0 are obtained by topological sorting algorithm. Then the GetNodeCandidate algorithm is called to obtain the candidate node *Cand*_*us*_ of the starting matching node *u*_*s*_, and the above steps are shown in lines 1-2 of Algorithm 2. It loops through the candidate node set of the starting node, and then uses the pattern edge matching methods EdgeMatching and EdgeAttribute to match all matching paths that meet the conditions, and then calls the Recursivematching method to obtain the matching subgraph *G*_*sub*_, and adds the matching subgraph to the matching subgraph set $G_{sub}^{All}$. This is shown in lines 4-10 of Algorithm 2.

**Algorithm 2:** TEM algorithm

Require: Data graph *G*_*D*_ and Pattern graph *G*_*P*_
Ensure: Set of all matching subgraphs $G_{sub}^{All}$
Get the topological sorting sequence *V*_*T*_ of the pattern graph and the node *V*_*E*_ with zero degree
Call GetNodeCandidate algorithm to get candidate node *Cand*_*us*_, which starts with matching node *u*_*s*_
Example Initialize the storefile,i = 0, num=0
while i < length(*Cand*_*us*_) do
*v*_*s*_=*Cand*_*us*_[i]
Edge(*v*_*s*_,v')=EdgeMatching(*v*_*s*_,(*u*_*s*_,u'))
*G*_*temp*_=EdgeAttribute(Edge(*v*_*s*_,v'))
if There is a node corresponding to the start node of the pattern edge then
*G*_*sub*_=Recursivematching(num+1,*V*_*T*_,*V*_*E*_,storefile)
end if
Add the intermediate result *G*_*sub*_ to $G_{sub}^{All}$
i=i+1
end while
return $G_{sub}^{All}$

The specific execution steps of the Recursivematching method are shown in Algorithm 3. Firstly, it is determined whether the number of the currently processed pattern edge is equal to the number of edges of the pattern edge. If it is equal, it means that all pattern edges have been processed and the matching results can be judged and stored, as shown in lines 1-5 of Algorithm 3. If not, loop through the topological sort nodes and match the pattern edges for each candidate node, as shown in lines 7-16 of Algorithm 3. The result that has been matched is read from the cache, and if there is no one in the cache, the GetNodeCandidate function is called to obtain the candidate node set *Cand*_*uc*_ of *u*_*c*_, as shown in lines 9-12 of Algorithm 3. For each candidate node *v*_*c*_, EdgeMatching and EdgeAttribute are called to filter edges with multiple constraints and match the next edge recursively, as shown in lines 13-16 of Algorithm 3.

**Algorithm 3:** Recursivematching algorithm.

Require: The number of the pattern edge num, the topological sort sequence *V*_*T*_ and the node *V*_*E*_ with zero degree
Ensure: The result storefile
if num=the number of sides of the pattern graph **then**
for All nodes x of the pattern graph do
Clear the matching nodes and edges that do not meet the conditions
Store the matching result *G*_*temp*_ to storefile
end for
return
end if
i=1
while i < length(*V*_*T*_) do
*u*_*c*_=*V*_*T*_[i]
if *u*_*c*_ ∈ *V*_*E*_ then
Call the GetNodeCandidate function to get the candidate node set *Cand*_*uc*_ for *u*_*c*_
elseRead results *Cand*_*uc*_ from *G*_*temp*_
end if
for Each candidate node *v*_*c*_ in *Cand*_*us*_ do
Edge(*v*_*c*_,v')=EdgeMatching(*v*_*c*_,(*u*_*c*_,u'))
*G*_*temp*_=EdgeAttribute(Edge(*v*_*c*_,v'))
end for
Recursivematching(num+1,*V*_*T*_,*V*_*E*_,storefile)
end while
return storefile

Example 3.1. Taking the pattern graph in Figure 1 and the data graph in Figure 2 as examples, the TEM algorithm firstly sorts the nodes in the pattern graph shown in Figure 1, and obtains the topologically ordered sequence *V*_*T*_ ={AP,PM,TC,TI,TM} of pattern nodes and the set *V*_*E*_ ={AP,PM} of pattern nodes with in-degree 0. Then, the set of candidate nodes *Cand*_*AP*_={Helen} of AP is obtained. Helen is added to the list of AP candidate nodes in *G*_*temp*_, and the pattern edge (AP,TI) matching starts from Helen. After the matching path (Helen,RATS) is obtained, the candidate node set *Cand*_*TI*_={RATS} of pattern node TI is also obtained. Then, starting from the second node of the pattern node topological sorting sequence *V*_*T*_, the pattern nodes are matched in turn according to the topological sequence of the nodes in the pattern graph.

**Figure 4:**
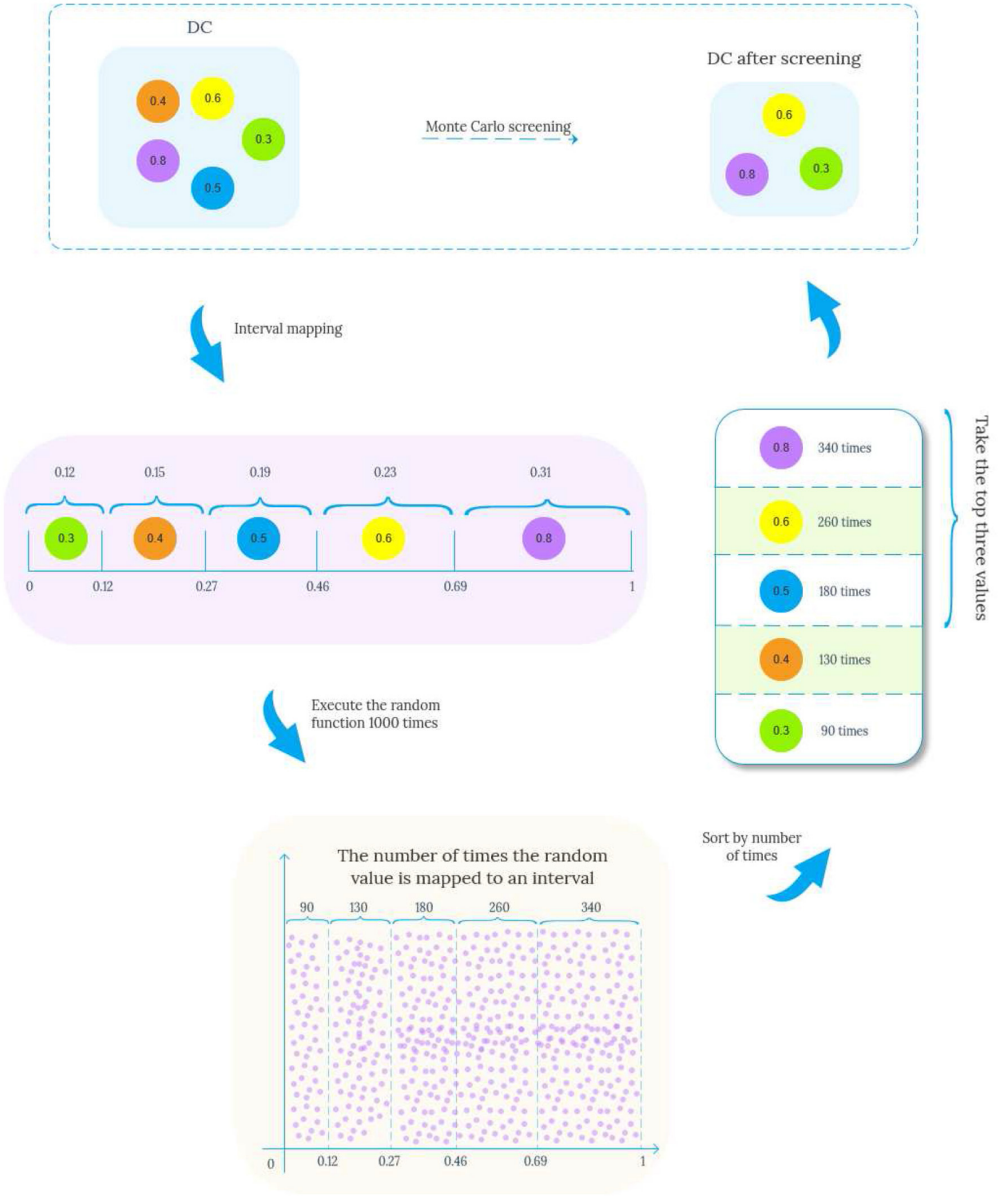
The screening process of DC.

The general idea of Algorithm 4 is to start from node *v*, breadth-first traverse the edges in the data graph, and record each traversal path $path_{j}\left(v,v^{\prime }\right)$ starting from *v*. When the end point of the path *v*′ matches *u*′, $path_{j}\left(v,v^{\prime }\right)$ is added to the set of matching paths *Edge*(*v, v*′) satisfying the path length constraint. After obtaining the matching paths of all full conditions, the EdgeAttribute method needs to be called again to review the multiple constraints defined on the pattern edge (*u, u*′), and the specific steps are shown in Algorithm 5. For a list of paths from *v* to *v*′ *pathlist*(*v, v*′), compute the aggregated values *A*_*DT*_,*A*_*DC*_,*A*_*CV*_ on each path, and then perform Monte Carlo filtering on the attributes *T*_*DC*_ and *T*_*CV*_ on the edges. Taking the screening of *T*_*DC*_ as an example, the specific process is shown in Figure 4. The proportion value *k* for each node is obtained by normalizing each attribute value by dividing it by the sum. These values *k* are then mapped onto an interval f of [0, 1], proportional to its size. Generate multiple random values in the range [0, 1] by repeatedly executing a random function. The generated values are mapped to intervals *f* and the proportion of values mapped to each interval is calculated, selecting the attribute value corresponding to the top *n* (adjustable parameter) values that exhibit a significant proportion as the new attribute value, as shown in lines 3-13 of Algorithm 5. Subsequently, the range of constraint values is judged, and the paths that conform to the constraint values are added to *G*_*temp*_.

**Algorithm 4:** EdgeMatching algorithm.

Require: Pattern edge (u,u'), start node v ∈ V and data graph *G*_*D*_
Ensure: Set Edge(v,v') that satisfies the path length constraint
Initialization Q = ∅
while Queue Q is not empty do
Take a path from Q pathj(v,v')
if *path*_*j*_(v,v') is not in Edge(v,v') then
if There is another path from v to v' in Edge(v,v') then
Add *path*_*j*_(v,v') to pathlist(v,v')
elseAdd *path*_*j*_(v,v') to Edge(v,v')
end if
end if
end while
Obtain adjv, the set of adjacent nodes of node v
for L in adjv do
if L satisfies the length constraint and L satisfies the constraint defined on the node u' then
pathi=*path*_*j*_(v,v') + (v,adjv)
Add pathi to Q
end if
end for
return Edge(v,v')

**Algorithm 5:** EdgeAttribute algorithm.

Require: Edge(v,v'),$f_{E}^{P}$(u,u'),$f_{m}^{P}$
Ensure: *G*_*temp*_
while For each v' in Edge(v,v') that satisfies the length constraint, the pathlist pathlist(v,v') do
*totalSum*_*DC*_=0
*totalSum*_*CV*_=0
while For every path P in pathlist(v,v') do
Calculate aggregate values *A*_*DT*_,*A*_*DC*_and*A*_*CV*_ for each attribute
*totalSum*_*DC*_+=$A_{DC}\left(v,v^{\prime }\right)$
*totalSum*_*CV*_+=$A_{CV}\left(v,v^{\prime }\right)$
end while
Calculate the proportion of each value in *totalSum*_*DC*_ and *totalSum*_*CV*_, and use the random function to map into the interval [0,1] to obtain *f*_*DC*_ and *f*_*CV*_.
for i do from 1 to 1000
Record the number of times mapped to each interval in *f*_*DC*_ *second*_*DC*_;
end for
for j do from 1 to 1000
Record the number of times mapped to each interval in *f*_*CV*_ *second*_*CV*_;
end for
*second*_*DC*_ and *second*_*CV*_ are ranked separately
The first n second values with large values and a crossing point for this path are taken as the candidate path pathlist2
while F door each path P in pathlist2(v,v')
if $\;\text{then}f_{DT}^{m}\left(A_{DT}\left(V,V^{\prime }\right)\right)$ ≥ Dtm,$f_{DC}^{m}\left(A_{DC}\left(V,V^{\prime }\right)\right)$ ≥ DCm,$f_{CV}^{m}\left(A_{CV}\left(V,V^{\prime }\right)\right)$ ≥ CVm
SuitP(v,v')=P
end if
end while
if S thenuitP(v,v') is not null
add SuitP(v,v') to *G*_*temp*_
end if
end while
return *G*_*temp*_

Example 3.2. For matching paths e1(SLCLIA,Drug), e2(SLCLIA,Operation) and e3(SLCLIA,Drug,Operation) of edge (TC,TM) in pattern Figure 1, where Φ={*A*_*DT*_,*A*_*DC*_,*A*_*CV*_}, Φ represents the set of aggregated attribute values on the path, Φ_*e*_1__ = {0.5, 0.7, 0.5}, Φ_*e*_2__ = {0.5, 0.6, 0.6}, Φ_*e*_3__ = {0.5, 0.48, 0.54}. Suppose we want to select two candidate edges at random, then we first sum the *T*_*DC*_ and *T*_*CV*_ of the three edges to obtain *totalSum*_*DC*_ = 1.78, *totalSum*_*CV*_ = 1.64, Then the proportion of *T*_*DC*_ and *T*_*CV*_ of the three edges in *totalSum*_*DC*_ and *totalSum*_*CV*_ is *P*_*e*1_=(0.39, 0.3), *P*_*e*2_=(0.34, 0.37), *P*_*e*3_=(0.27, 0.3), respectively. Similarly, we can get the mapping interval *f*_*DC*_ = [e1 (0-0.39), e2 (0.4-0.73), e3 (0.74-1)] and CV mapping interval *f*_*CV*_ = [e1 (0-0.3), e2 (0.31-0.67), e3 (0.68-1)]. Executing the random function from 0 to 1,000 times yields the number of times it maps to the two interval values *second*_*DC*_=(431, 330,239), *second*_*CV*_=(378,511,111), assuming the first two largest numbers. Then path e1 and path e2 enter the next step as candidate paths for normal edge condition matching to filter out edges with too low constraint value.

### 3.2 Description of the THM algorithm

THM algorithm is a combination of LcKG-McGPM algorithm and TEM algorithm, and Monte Carlo method is applied in node matching and edge matching. Therefore, the main difference between the THM algorithm and the TEM algorithm lies in the screening of candidate nodes, that is, the difference in the GetNodeCandidate function. The rest of the part is not discussed here, please refer to Algorithm 6.

**Algorithm 6:** GetNodeCandidate algorithm.

Require: Node whose mode is to be matched u ∈ *V*_*P*_, Node set V of the data graph
Ensure: The set of candidate nodes *Cand*_*u*_ of *u*
while For each node V in v, if V.ited =false do
if *label*_*v*_(u) ∈ *label*_*v*_(v) then
Add *v* to *u*'s set of candidate nodes, nodeCandidate
end if
end while
Example Initialize totalSum=0
for *u*_*i*_ in nodeCandidate do
totalSum+=*u*_*i*_.factor
end for
The proportion of each value is calculated and mapped to the interval [0,1] using the random function to get ratioValues
for i from 1 to 1,000 do
Records the number of times mapped to each value in ratioValues (second)
end for
Sort the second
Add the first n second largest nodeCandidate to *Cand*_*u*_
return *Cand*_*u*_

## 4 Experiments

In this section, our experiments were conducted using a PC running Windows 10, equipped with an Intel Core i9-10900F CPU clocked at 2.80 GHz and 32 GB of RAM. In order to ensure the authenticity and fairness of the experimental data, the data sets used in this paper are LCKGdataset1, LCKGdataset2 and LCKGdataset3 created by Li et al. (2024). Each dataset possesses a unique edge and node configuration with detailed specifications shown in Table 1. To reduce potential errors and ensure reliability, the reported results are arithmetic averages from ten iterations of each graph with different configurations.

**Table 1:** Data sets adopted in experiments.

**Data set**	**Number of nodes**	**Number of edges**	**Descriptive information**
LCKGdataset1	6,020	178,414	Tumor knowledge graph data set 1
LCKGdataset2	75,000	934,577	Tumor knowledge graph data set 2
Slashdot	77,360	905,468	A friend/foe social network
LCKGdataset3	78,000	949,289	Tumor knowledge graph data set 3

**Figure 5:**
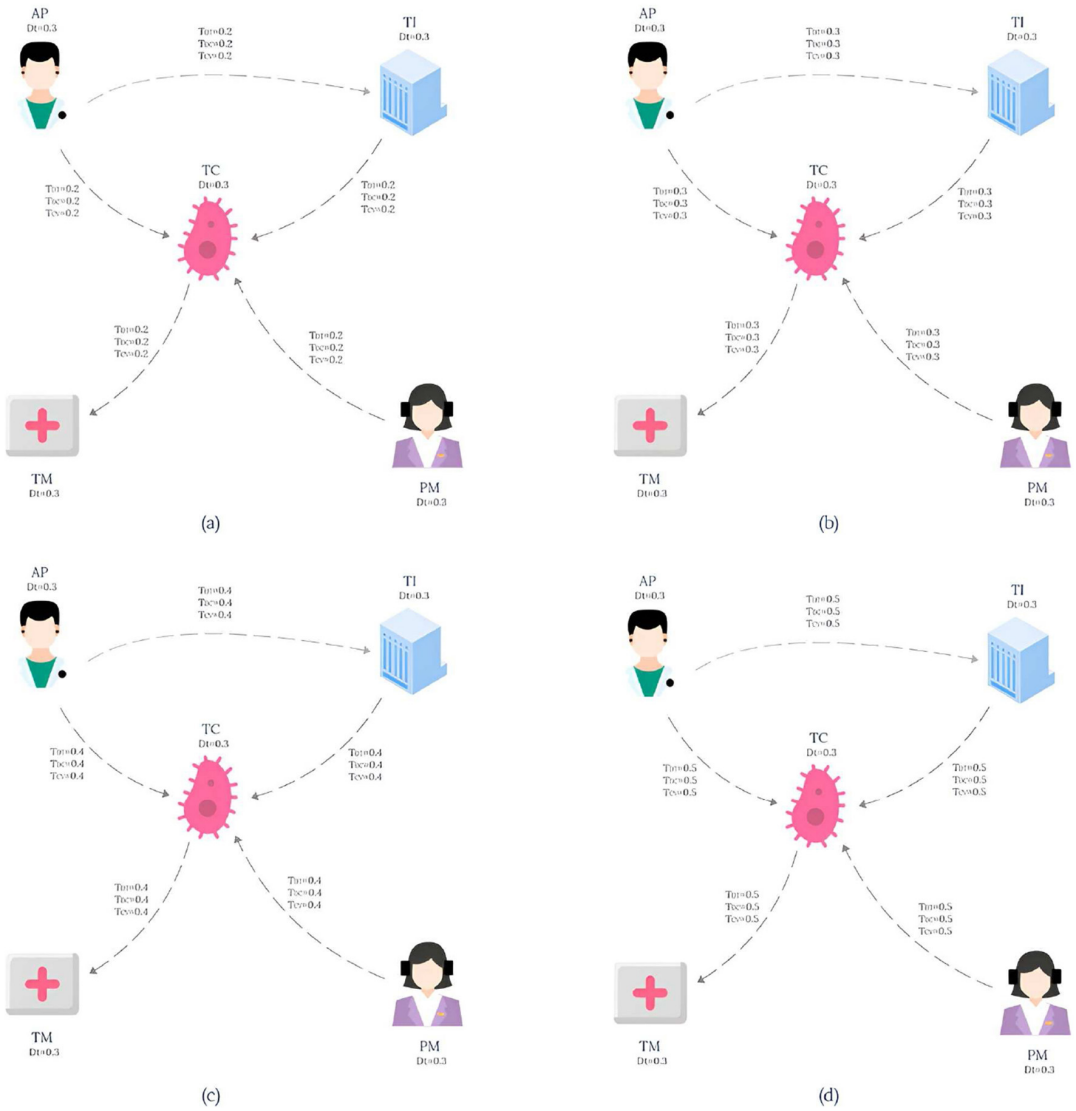
Pattern graph for the experiment.

**Figure 6:**
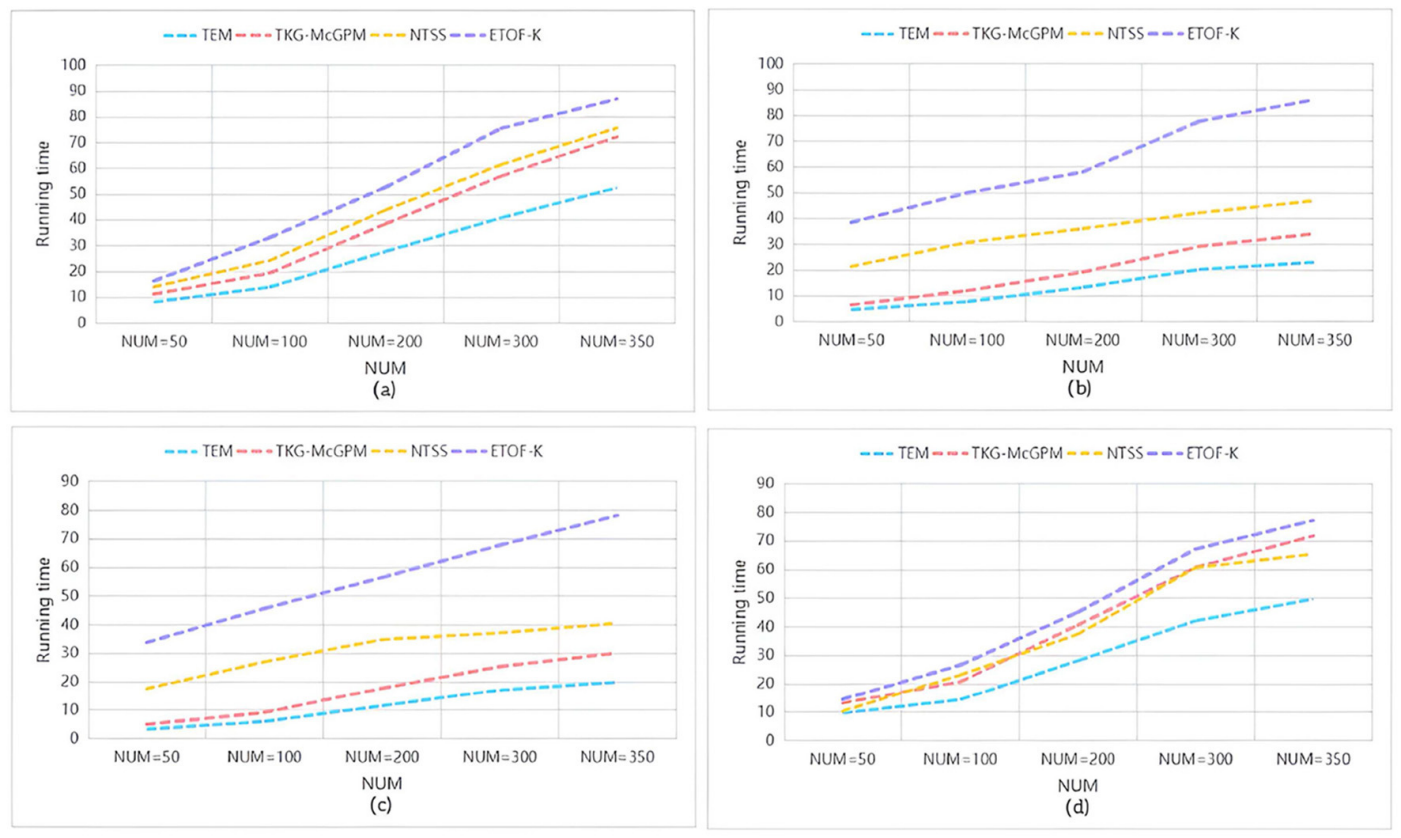
The change in the time taken to return different numbers of matching subgraphs on different data sets.

To compare the time efficiency of various algorithms on different datasets, we calculated the duration required for each algorithm to return an equal number of matched subgraphs, and the results are shown in Figure 5. Here, the vertical axis represents the execution time of the algorithm (in seconds), while the horizontal axis represents the number of matched subgraphs (NUM) , and from (a) to (d) represent the results of running on the datasets LCKGdataset1, LCKGdataset2, Slashdot, and LCKGdataset3, respectively. From Figure 6, it is obvious that no matter how much NUM is controlled, the time required by our proposed TEM algorithm is significantly less than that of the TKG-McGPM algorithm, that is, the time efficiency of the TEM algorithm is better than that of the TKG-McGPM algorithm.

In the complex process of graph pattern matching, a key factor lies in the characteristic of graph structure itself, that is, the number of edges is usually smaller than the number of nodes. Especially in the four datasets selected in this experiment (LCKGdataset1, LCKGdataset2, Slashdot and LCKGdataset3), this phenomenon of quantitative difference is particularly prominent. For example, in the process of running on LCKGdataset1, after detailed statistics and analysis, the number of nodes and edges involved in matching reaches thousands, while the number of edges is only about half of the number of nodes. The same is true for several other datasets, which means that graph pattern matching is theoretically less computationally expensive for edges than for nodes.

Our TEM algorithm focuses on the Monte Carlo method applied to the edge matching process, while the TKG-McGPM algorithm uses relevant mechanisms and involves more computational considerations in the node matching process. Since Monte Carlo method itself is based on random sampling to operate, each operation will incur a corresponding computational cost. When dealing with nodes, due to the large number of nodes, more elements need to be traversed and analyzed, which leads to the accumulation of computing time. As for edges, due to the relatively small number of edges, the number of random sampling, condition judgment and corresponding calculation operations will be reduced when using the Monte Carlo method, so the calculation time required by the Monte Carlo method for edges will be less than that for nodes.

Based on the above analysis, in the actual matching process, TEM algorithm can effectively avoid a large amount of computing consumption caused by too many nodes by virtue of its advantage of using Monte Carlo method in edge matching, so as to produce less time overhead. This reduction in time cost is not a small, local change, but plays a positive role in the entire matching process. From the overall point of view, this makes the whole matching process run more efficiently, and then improves the overall matching efficiency. It not only means that the results can be output faster in a single matching task, but also in the face of large-scale graph pattern matching requirements or scenarios that require multiple repeated matching operations, the time cost saved by TEM algorithm will be more considerable, which provides a more timely and feasible solution for practical applications.

**Figure 7:**
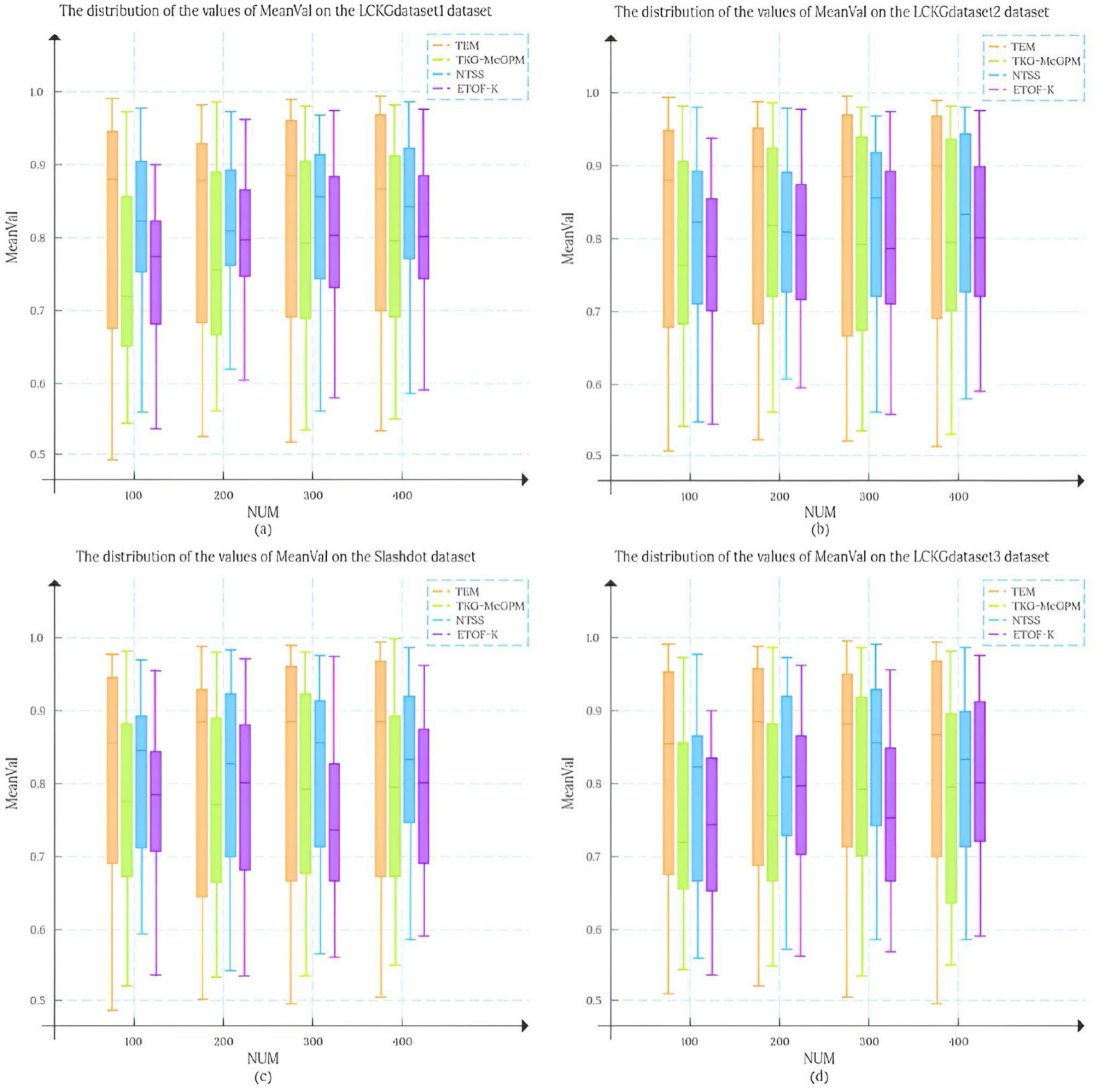
The distribution of MeanVal values on different data sets.

In this study, we set the number of matching results (NUM) to 100, 200, 300, and 400, respectively, and randomly draw 50 matching edges from each result set. To analyze the matching results more systematically, we conducted an in-depth analysis of the mean distribution of *T*_*DC*_ values and *T*_*CV*_ values, and this statistic is defined as MeanVal. As shown in Figure 7, the X-axis represents the number of matching results (NUM), and the Y-axis represents the mean MeanVal. Compared with the TKG-McGPM algorithm, our proposed TEM algorithm shows a wider distribution range, and most of the values are concentrated in the interval with high value ratios from 0.7 to 1.0.

Firstly, this shows that the key element of the diversity of matching results is fully taken into account in the design and operation of the TEM algorithm. In the actual application scenarios of graph pattern matching, the diversity of matching results means that users can be provided with more potential matching options with different perspectives and different characteristics, which is particularly important for complex application fields such as medical knowledge graphs. For example, in medical decision AIDS, diverse matching results can cover multi-dimensional information such as different treatment plans and different diagnostic ideas, so as to help medical personnel make more comprehensive considerations. The reason why TEM algorithm can achieve such diversity is that its internal mechanism is not limited to a single matching path or simple matching rule in the matching process, but through reasonable strategies, it can mine and present different possibilities from multiple levels when generating matching results, avoiding the homogenization of results. Therefore, the distribution range of the final MeanVal is broadened to cover a wider range of numerical intervals, especially in the high-value ratio interval, which shows a relatively concentrated trend, which also reflects that the algorithm can effectively filter out those matching edges with low relative value and focus on the matching results with more value and higher quality. The matching edge with high value can be highlighted in the result, and the reference value of the overall matching result is improved.

In contrast, NTSS and ETOF-K can also achieve high mean values in some cases and seem to provide good quality matching results on the surface, but a closer look at the distribution of their MeanVal shows a narrow distribution. This phenomenon is not accidental, and it profoundly reflects the characteristics of the strategies adopted by these algorithms in optimizing the matching results and the possible limitations. In the process of optimizing matching, these two algorithms focus more on the direct improvement of the quality of matching results, for example, they may try to make each output matching result as close as possible to a preset high quality standard by means of stricter screening rules and more accurate matching conditions. However, this approach to some extent makes them fall short in mining the diversity of potential matches. Because the algorithm focuses too much on the optimization of quality, it is easy to follow a relatively fixed and limited path to find results when searching the matching space, thus ignoring other potential matches that may exist, although they are not optimal in some indicators, but have unique value. Finally, the matching results show the characteristics of concentration and narrow range in the numerical distribution. It can not cover more possibilities as comprehensively as TEM algorithm, so it may provide relatively limited reference information in the face of complex and changing practical application requirements.

Further analyzing the root cause of this difference, for the TKG-McGPM algorithm, diversity is given higher importance in the node matching stage, which is worth recognizing, because as the key elements in the graph structure, the diverse matching combinations of nodes can bring rich changes to the whole matching results. However, the algorithm has some shortcomings in the edge matching link, which only considers the size of the comprehensive value of diagnosis and treatment. In the actual graph pattern matching, the edge not only carries simple numerical information, but also the connection relationship between the edge and the node, the topological structure position and other factors affect the diversity and rationality of the matching. However, the TKG-McGPM algorithm does not fully consider the influence of these edge-related multi-dimensional features on the matching diversity, which leads to the loss of many potential possibilities that can enrich the matching results in the process of edge matching, and then the overall matching results are limited in diversity.

**Figure 8:**
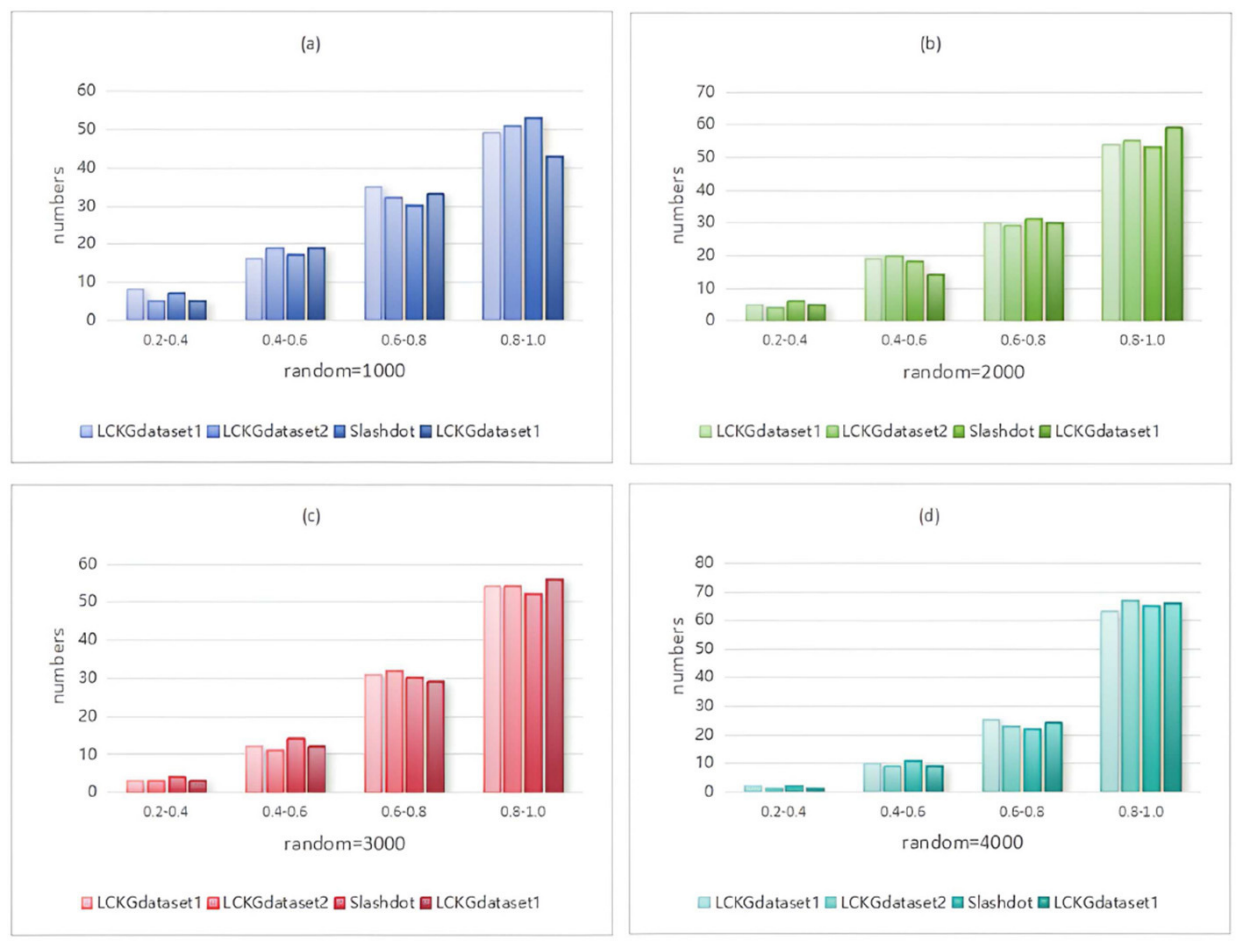
When NUM=100, different random executions on different datasets affect the distribution of constraint values on edges.

**Figure 9:**
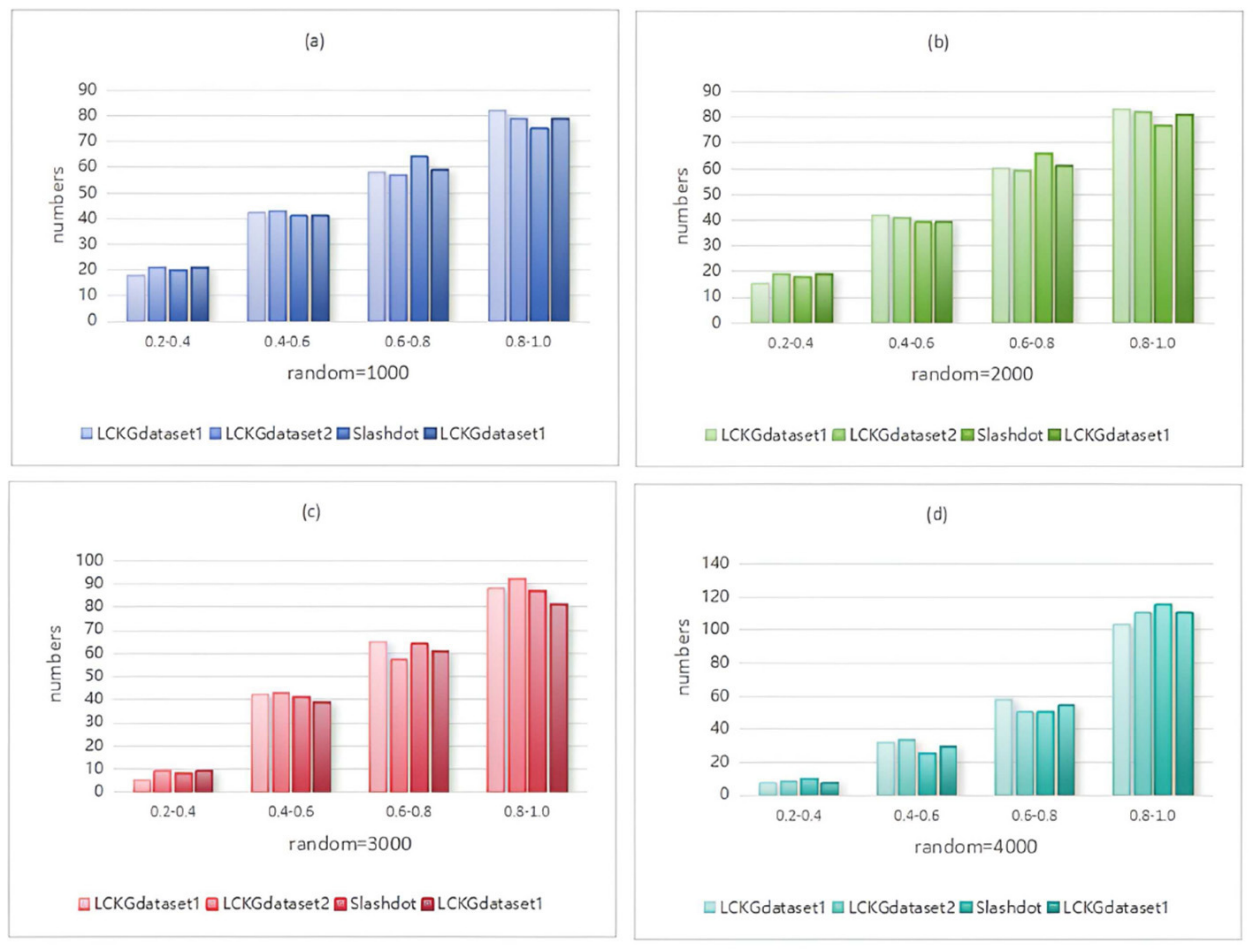
When NUM=200, different random executions on different datasets affect the distribution of constraint values on edges.

**Figure 10:**
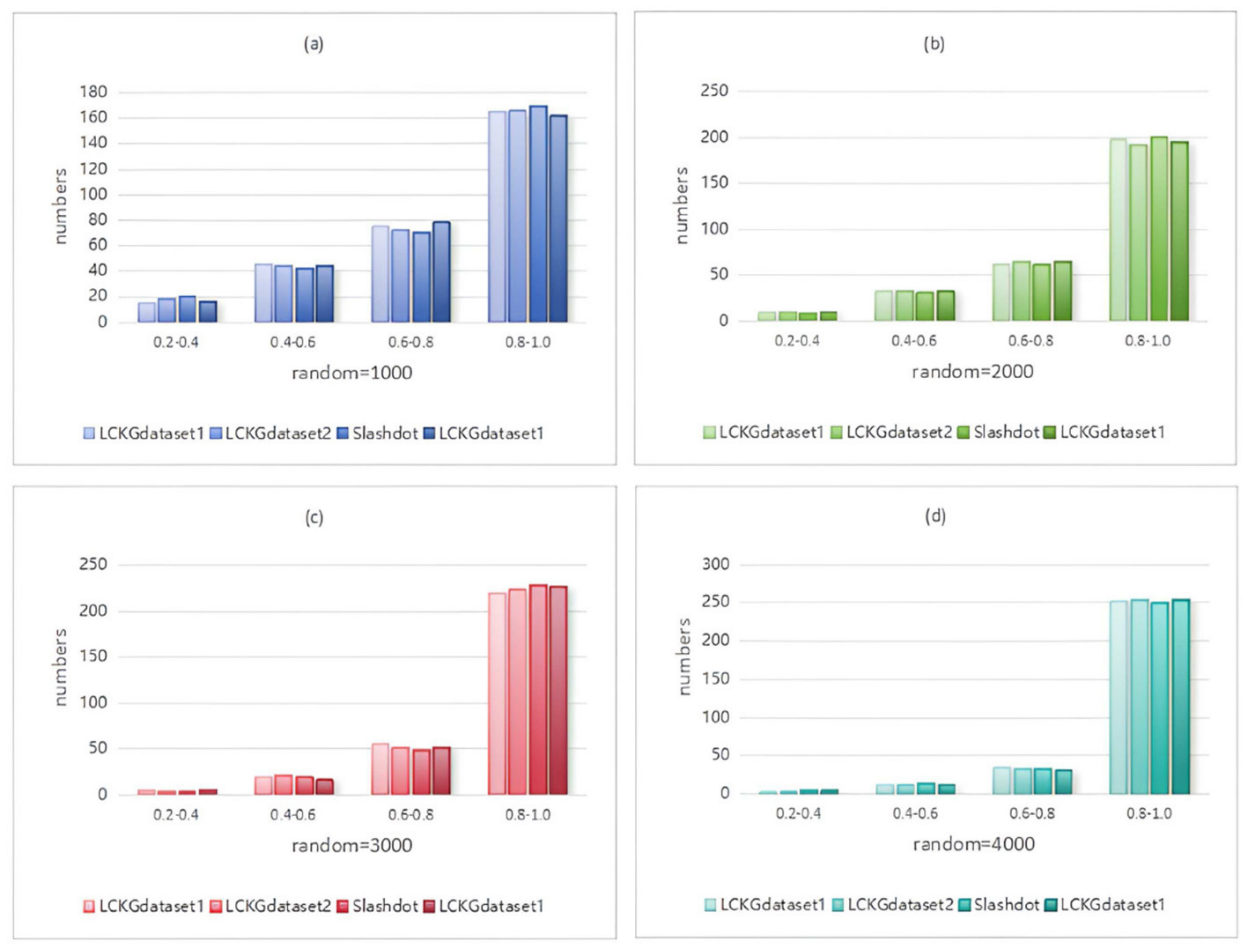
When NUM=300, different random executions on different datasets affect the distribution of constraint values on edges.

**Figure 11:**
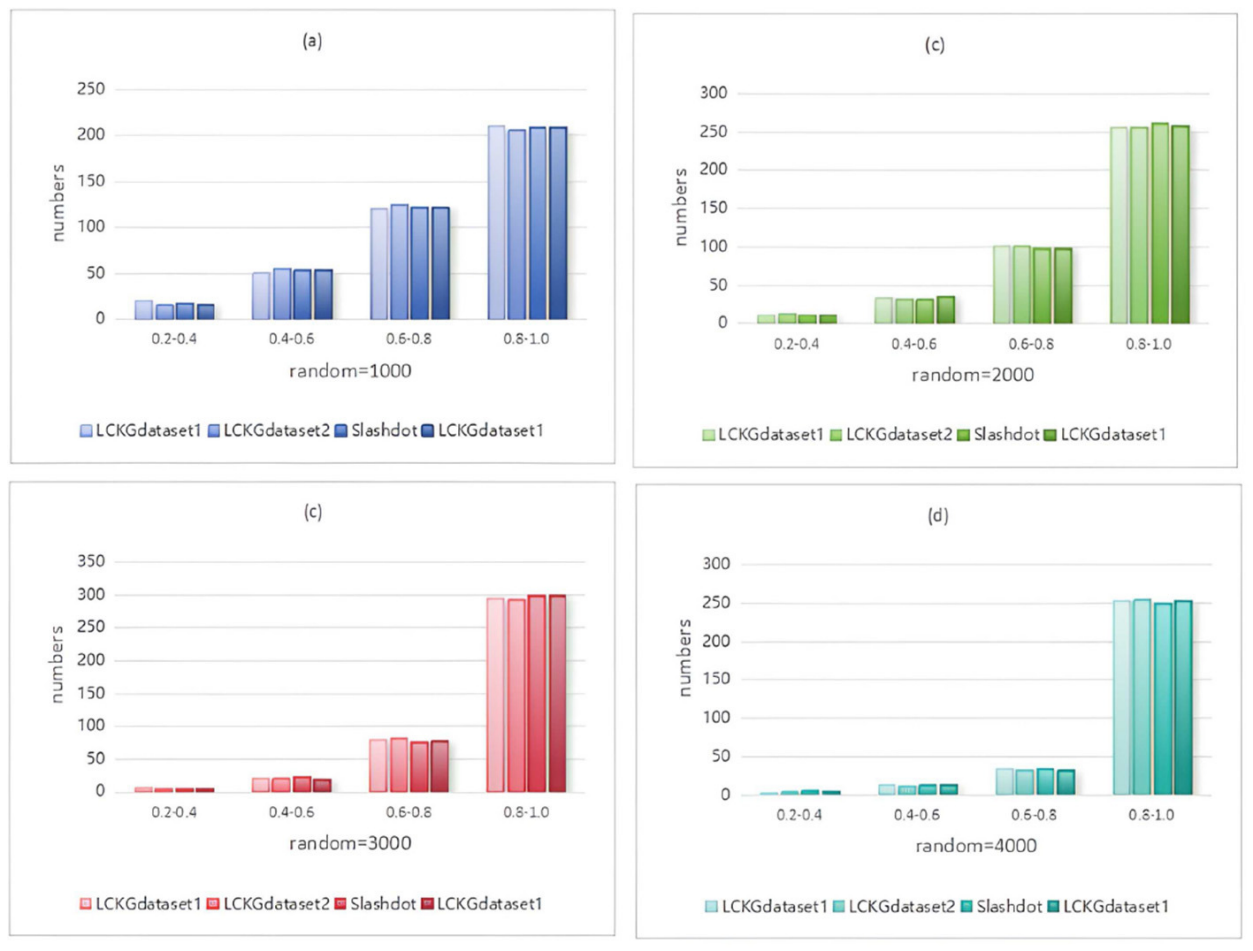
When NUM=400, different random executions on different datasets affect the distribution of constraint values on edges.

In Monte Carlo simulation, randomness is a key parameter factor that affects the performance of the algorithm. Immediately following, we analyze the effect of different random execution times on the distribution of constraint values on edges , as shown in Figures 8–11. The X-axis of each plot is the mean, MeanVal, and the Y-axis is the number of matched subplots.In Monte Carlo simulations, the range involved in random execution counts is determined by multiple experiments. Specifically, when the value of random execution count is too small, its impact on the final result is relatively weak. If the count is too large, it will undoubtedly cost more time. Based on several rigorous experimental explorations and comprehensive consideration of various factors, we carefully selected four relatively ideal counting results of 1,000, 2,000, 3,000, and 4,000 to carry out comparative experiments. Through such an experimental setting, the influence of random execution times on constraint values can be more intuitively presented, which is helpful for us to analyze the internal relationship between relevant variables deeply and accurately, so as to provide a strong basis for subsequent research and conclusion derivation. It can be seen from the figure that when controlling the number of matching results (NUM), the value of MeanVal tends to stabilize in a higher numerical interval as the number of random executions increases. This indicates that the increase of the number of random executions can improve the accuracy of the matching results, that is, the more random times, the closer the results are to the optimal solution. Therefore, we can satisfy patient preferences by providing different random number options. For patients with high matching level priority, more random samples can be selected to obtain matching results with higher *T*_*DC*_ and *T*_*CV*_ values. However, for patients who do not care much about the matching values on the edges, a lower random frequency can be chosen to ensure a more uniform distribution of *T*_*DC*_ and *T*_*CV*_ in the matching results.

From a macro point of view, this method of flexibly adjusting the number of random executions according to different patient preferences not only fully demonstrates the flexibility and adaptability of the matching algorithm, but also has important practical significance in practical applications. Its flexibility is reflected in the ability to quickly adjust the running parameters of the algorithm to generate matching results that meet specific requirements. Adaptability shows that the algorithm can be widely applied to different patient groups with different preference types. Whether patients focus on high-quality matching or balanced and stable matching results, the algorithm can effectively meet the needs. At the same time, by allowing patients to understand the credibility of the matching results more clearly and comprehensively, it further enhances the patient's acceptance and sense of identity for the whole matching process and the final result, which helps to improve the practicability and promotion value of the algorithm in the actual medical scene, so that it can better serve the patient groups with different needs. It provides strong support for medical decision-making and other related applications.

Considering the potential application of Monte Carlo method in node and edge matching, we combine the TKG-McGPM algorithm with the TEM algorithm, and propose the THM algorithm by using Monte Carlo method on both edges and nodes. In order to comprehensively and objectively evaluate the actual performance of THM algorithm, we carefully design and carry out a series of rigorous performance analysis work, focusing on the comparative analysis with TEM algorithm.

**Figure 12:**
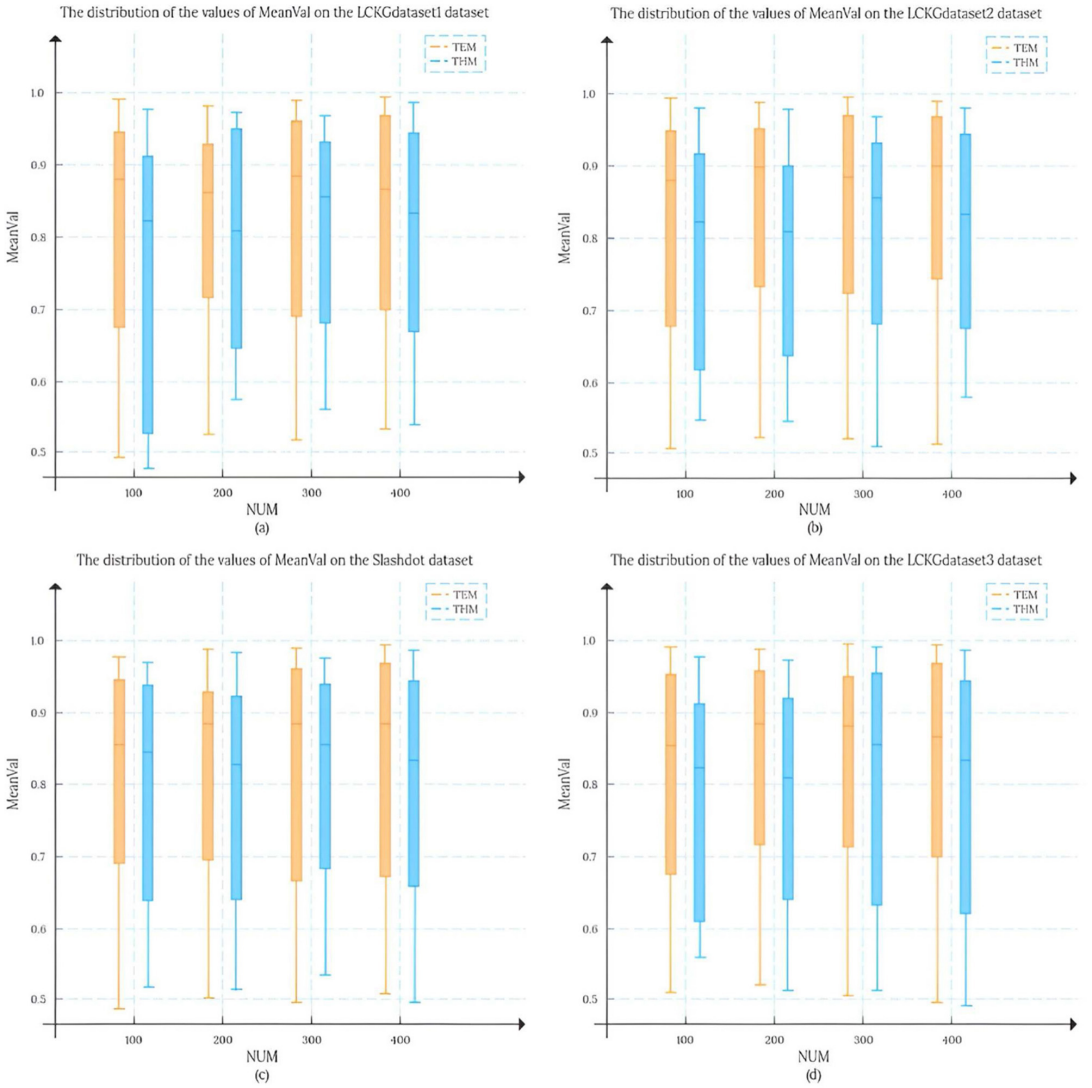
The distribution of MeanVal values between the two algorithms on different datasets.

**Figure 13:**
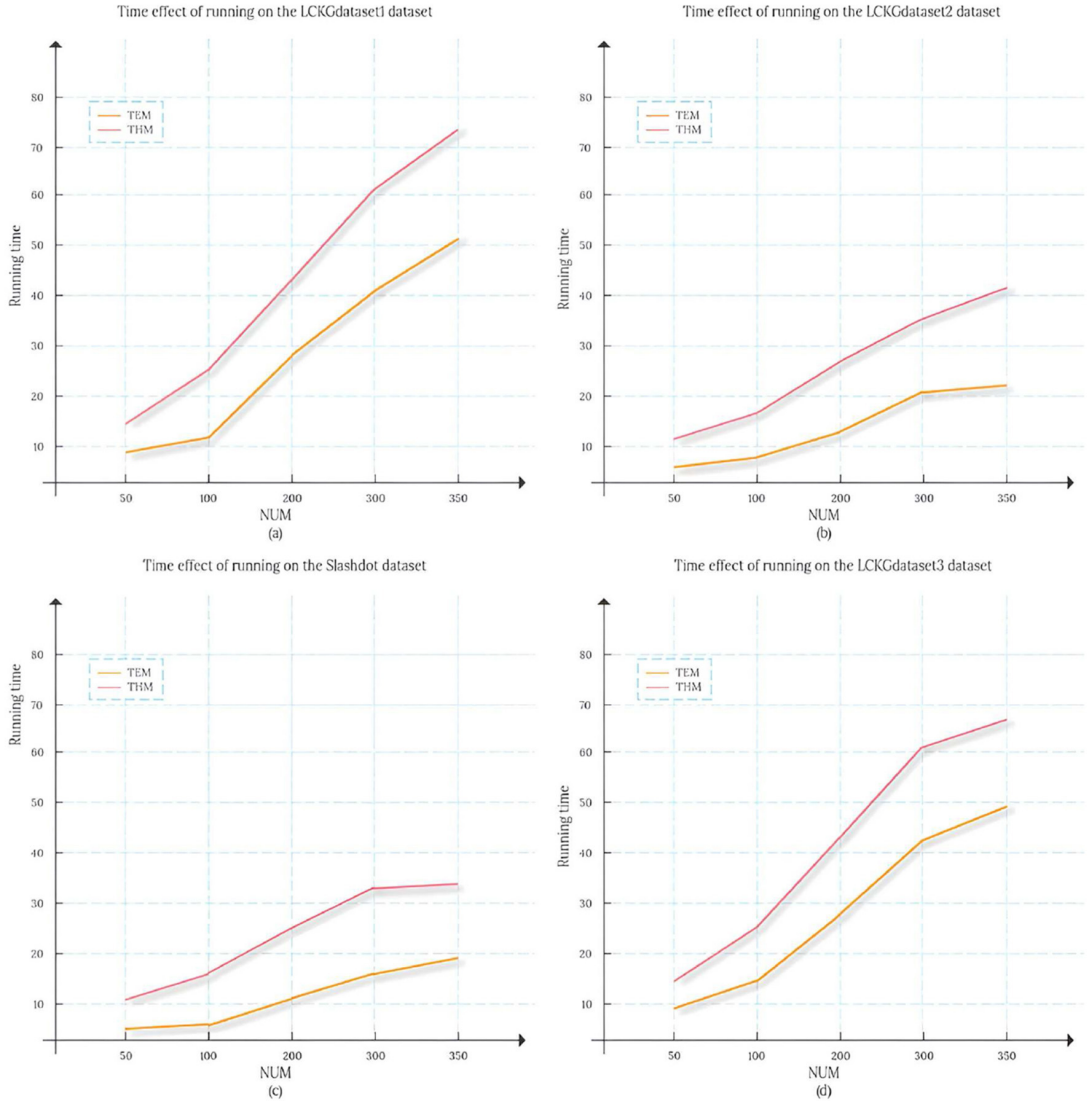
Temporal effects of running on different datasets.

The reason why TEM algorithm is selected as the comparison object is that TEM algorithm has shown certain advantages and characteristics in previous research and practical application. Through the comparison of the two, the characteristics, advantages and disadvantages of THM algorithm can be more clearly highlighted. Specifically, the THM algorithm is able to generate more diverse solutions by applying Monte Carlo methods on the nodes and edges, which is clearly demonstrated in Figure 12. We can see that compared with TEM algorithm, the matching results generated by THM algorithm show more abundant changes in the interval distribution of the mean MeanVal. However, any algorithmic feature always comes with a certain cost. For THM algorithm, its advantage of producing diverse solutions is at the cost of consuming a large amount of computing resources. For THM algorithm, its advantage of producing diverse solutions comes at the cost of consuming a lot of computing resources. The reason behind this is that the Monte Carlo method is essentially a random sampling-based method, and its working principle dictates that a large number of simulation operations are required in order to obtain reliable and statistically significant results. Each simulation sampling process involves the calculation and judgment of the attributes and relationships of nodes and edges. With the continuous accumulation of simulation times, the consumption of computing resources increases geometrically. Especially in the face of large-scale medical knowledge graphs or complex graph structures, the consumption of such computing resources will be more significant. For example, when dealing with the actual medical knowledge graph containing massive medical data nodes and complex edge relationships, the THM algorithm may need to occupy a large amount of memory space for storing intermediate calculation results. At the same time, the processor also needs to be in a high load state for a long time to complete numerous simulation sampling and result statistics.It is this high dependence on computing resources and large consumption that directly leads to the obvious disadvantage of THM algorithm in terms of time efficiency, which is clearly presented in Figure 13.

Considering the above factors such as time efficiency and practicality of results, although THM algorithm has a non-negligible advantage in diversity, TEM algorithm has undoubtedly become a more ideal choice due to its excellent performance in these key dimensions. It can better balance the relationship between algorithm performance and practical application requirements, provide efficient and practical solutions for graph pattern matching tasks in medical knowledge graph and other related fields, and better meet the current requirements for fast response and accurate decision-making in practical applications of algorithms.

## 5 Conclusions

In the context of current research on graph pattern matching algorithms, this study takes the existing KG-McGPM algorithm as the cornerstone, and through in-depth analysis and innovative exploration, proposes two improved graph pattern matching algorithms: TEM and THM, which are mainly used in subgraph matching of lung cancer knowledge graph. By introducing the DC and CV parameters on the edge of the Monte Carlo method, the diversity of matching results is increased. In order to improve the matching effect, the original graph pattern matching model has been modified, and a marginal graph pattern matching algorithm based on lung cancer knowledge graph (TEM) has been proposed. To further verify the effectiveness and efficiency of TEM, a hologram pattern matching algorithm (THM) has been proposed, and the Monte Carlo method has been applied to nodes and edges. Experimental results show that the performance of the TEM algorithm is better than that of the existing algorithms and the THM algorithm. Although the algorithm proposed in this study has achieved excellent performance in the specific field of lung cancer knowledge graph, there is still room for further exploration and improvement from the perspective of macro academic research and practical application expansion. Future research work can focus on extending these algorithms to other medical fields and different types of knowledge graph application scenarios, and deeply investigate their versatility and adaptability through practical verification in diverse contexts. This not only helps to further verify the scientific and reliability of the algorithm itself, but also provides an effective solution for graph pattern matching problems in more fields.

In addition, with the continuous development of computer technology and the increasing scale of data, the calculation speed and matching quality of the algorithm are always the key aspects that need to be paid attention to. Future research can focus on adopting more efficient data structures, such as exploring new graph storage structures or index methods, to optimize the efficiency of the algorithm in data storage and reading. At the same time, combined with advanced optimization algorithms, such as greedy algorithm, dynamic programming algorithm and other ideas, the calculation process of the existing algorithm is deeply optimized, and the overall calculation speed is effectively improved.

## Data Availability

The original contributions presented in the study are included in the article/supplementary material, further inquiries can be directed to the corresponding author.

## References

[B1] Carletti V. Foggia P. Saggese A. Vento M. (2018). Challenging the time complexity of exact subgraph isomorphism for huge and dense graphs with VF3. IEEE Trans. Pattern Analy. Mach. Intellig. 9, 804–818. 28436848 10.1109/TPAMI.2017.2696940

[B2] Cheng J. Yu J. X. Ding B. Yu P. S. Wang H. (2008). “Fast graph pattern matching,” in IEEE 24th International Conference on Data Engineering (Cancun: IEEE), 913–922.

[B3] Chikhaoui B. Tshimula J. M. Wang S. (2020). “Community mining and cross-community discovery in online social networks,” in International Conference on Network-Based Information Systems, 8. Available at: https://api.semanticscholar.org/CorpusID:221218843 (accessed January 30, 2025).

[B4] Fan W. Li J. Ma S. Tang N. Wu Y. Wu Y.. (2010). Graph pattern matching: from intractable to polynomial time. Proc. VLDB Endow. 3, 264–275. 10.14778/1920841.1920878

[B5] Fan W. Wang X. Wu Y. (2013). Incremental graph pattern matching. ACM Trans. Database Syst. 38:47. 10.1145/2508020.2489791

[B6] Foggia P. Sansone C. Vento M. (2001). “An improved algorithm for matching large graphs,” in Proc of the 3rd IAPR-TC-15 International Workshop on Graph-based Representation, Ischia, Italy (Ischia: Springer), 1–8.

[B7] Guo Z. (2024). Research on Quality Prediction of Continuous Casting Billet Based on Graph Pattern Matching. Beijing: *Metallurgical Automation Research and Design Institute*.

[B8] Henzinger M. R. Henzinger T. A. Kopke P. W. (1995). “Computing simulations on finite and infinite graphs,” in IEEE 36th Annual Foundations of Computer Science (Milwaukee, WI: IEEE), 453–462. 10.1109/SFCS.1995.492576

[B9] Hu J. Ferguson A. L. (2016). Global graph matching using diffusion maps. Data Analy. 20, 637–654. 10.3233/IDA-160824

[B10] Jin H. Huo H. Fang T. (2023). Strong simulation matching of temporal pattern graph with temporal priority constraints. Comp. Technol. Dev. 06, 88–94.

[B11] Khan A. Golab L. Kargar M. Szlichta J. Zihayat M. (2020). Compact group discovery in attributed graphs and social networks. Inform. Proc. Managem. 57, 0306–4573. 10.1016/j.ipm.2019.102054

[B12] Li L. He J. Wang M. Wu X. (2016). Trust agent-based behavior induction in social networks. IEEE Intellig. Syst. 31, 24–30. 10.1109/MIS.2016.6

[B13] Li L. Tu H. Tao Z. Bu C. Wu X. (2024). “Probabilistic graph pattern matching via tumor knowledge graph,” in ACM Transactions on Probabilistic Machine Learning (New York: ACM).

[B14] Liu F. Xue S. Wu J. Zhou C. Hu W. Paris C.. (2021). “Deep learning for community detection: progress, challenges and opportunities,” in Twenty-Ninth International Joint Conference on Artificial Intelligence (IJCAI'20) (Yokohama: IJCAI), 4981–4987. 10.24963/ijcai.2020/693

[B15] Liu G. Li L. Liu G. Wu X. (2022). Social group query based on multi-fuzzy-constrained strong simulation. ACM Trans. Knowl. Discov. Data 16:27. 10.1145/3481640

[B16] Liu G. Li L. Wu X. (2020). Multi-fuzzy-constrained graph pattern matching with big graph data. Intell. Data Anal. 24, 941–958. 10.3233/IDA-194653

[B17] Liu G. Zheng K. Wang Y. Orgun M. A. Liu A. Zhao L.. (2015). “Multi-Constrained graph pattern matching in large-scale contextual social graphs,” in IEEE 31st International Conference on Data Engineering, Seoul, Korea (Seoul: IEEE), 351–362.

[B18] Sato R. Habe H. Mitsugami I. Satake S. Sumi K. Yagi Y. (2016). Social group discovery extracting useful features using multiple instance learning. J. Japan Soc. Fuzzy Theory Intellig. Inform. 28, 920–931. 10.3156/jsoft.28.920

[B19] Su X. Xue S. Liu F. Wu J. Yang J. Zhou C.. (2022). A comprehensive survey on community detection with deep learning. IEEE Trans. Neural Netw. Learning Syst. 09, 2162–2388. 10.1109/TNNLS.2021.3137396 35263257

[B20] Tian Y. Patel J. M. (2008). “A tool for approximate large graph matching,” in 2008 IEEE 24th International Conference on Data Engineering (Cancun: IEEE), 963–972.

[B21] Tong H. Faloutsos C. Gallagher B. Rad T. E. (2007). “Fast best-effort pattern matching in large attributed graphs,” in 13th ACM SIGKDD International Conference on Knowledge Discovery and Data Mining (KDD '07), 737–746. 10.1145/1281192.1281271

[B22] Wei H. Yu J. X. Lu C. Jin R. (2014). Reachability querying: an independent permutation labeling approach. Proc. VLDB Endow. 7, 1191–1202. 10.14778/2732977.2732992

[B23] Yan M. (2023). Research on Multi-Constrained Graph Pattern Matching for Large Graph Data (MA thesis). Hefei University of Technology, Hefei, China.

