# Erratum: Edge-level multi-constraint graph pattern matching with lung cancer knowledge graph

**DOI:** 10.3389/fdata.2025.1582619

**Published:** 2025-03-04

**Authors:** Frontiers Production Office

**Affiliations:** Frontiers Media SA, Lausanne, Switzerland

**Keywords:** graph pattern matching, probability graph, lung cancer knowledge graph, Monte Carlo method, multi-constraint

Due to a production error, the DOI was written as 2024 instead of 2025. The correct DOI is: 10.3389/fdata.2025.1546850

The collection date in the XML should be: 2025.

The title of the article should be changed from: “*Edge-level multi-constranint graph pattern matching with lung cancer knowledge graph*” to “*Edge-level multi-constraint graph pattern matching with lung cancer knowledge graph*”.

The publisher apologizes for these mistakes. The original version of this article has been updated.

